# *Panax notoginseng* stems and leaves affect microbial community and function in cecum of duzang pigs

**DOI:** 10.1093/tas/txad142

**Published:** 2024-01-22

**Authors:** Lanlan Yi, Junhong Zhu, Qiuyan Li, Xuancheng Guan, Wenjie Cheng, Yuxiao Xie, Yanguang Zhao, Sumei Zhao

**Affiliations:** Yunnan Key Laboratory of Animal Nutrition and Feed Science, Yunnan Agricultural University, Yunnan 650201, China; Yunnan Key Laboratory of Animal Nutrition and Feed Science, Yunnan Agricultural University, Yunnan 650201, China; Yunnan Key Laboratory of Animal Nutrition and Feed Science, Yunnan Agricultural University, Yunnan 650201, China; Yunnan Key Laboratory of Animal Nutrition and Feed Science, Yunnan Agricultural University, Yunnan 650201, China; Yunnan Key Laboratory of Animal Nutrition and Feed Science, Yunnan Agricultural University, Yunnan 650201, China; Yunnan Key Laboratory of Animal Nutrition and Feed Science, Yunnan Agricultural University, Yunnan 650201, China; College of Biology and Agriculture, Zunyi Normal University, Guizhou 563006, China; Shanghai Academy of Science Technology, Shanghai Lab. Animal Research Center, Shanghai 201203, China; Yunnan Key Laboratory of Animal Nutrition and Feed Science, Yunnan Agricultural University, Yunnan 650201, China

**Keywords:** 16S rRNA sequencing, cecal, Duzang pig, metagenomic sequencing, *Panax Notoginseng* stems and leaves, short-chain fatty acids

## Abstract

*Panax notoginseng* is a Chinese medicine with a long history in which stems and leaves are the wastes of processing *Panax notoginseng* and have not been effectively utilized. The effects of diets containing *Panax notoginseng* stems and leaves on the cecal short-chain fatty acid (SCFA) concentration and microbiome of independent pigs were studied. Diets containing *Panax notoginseng* stems and leaves did not affect the concentration of SCFA in the cecal contents of Duzang pigs but affected the microbial composition and diversity. Firmicutes, Proteobacteria, and Bacteroidetes dominate in the cecal of Duzang pigs. Feeding Duzang pigs with a 10% *Panax notoginseng* stems and leaves diet increases the abundance of *Lactobacillus*, *Christensenellaceae R-7 group*, and *Akkermansia* in the cecal. We found 14 genera positively associated with acetate, and they were *Lactobacillus*, *Ruminococcaceae UCG 005*, *Ruminiclostridium 6; Escherichia Shigella* and *Family XIII AD3011 group* showed negative correlations. *Solobacterium*, *Desulfovibrio*, and *Erysipelatoclostridium* were positively associated with propionate. *Campylobacter*, *Clostridium sensu stricto 11*, and *Angelakisella* were positively associated with butyrate. In conclusion, *Panax notoginseng* stems and leaves could affect the cecal microbial community and functional composition of Duzang pigs. *Panax notoginseng* stems and leaves reduce the enrichment of lipopolysaccharide biosynthetic pathway of the cecal microbiome, which may have a positive effect on intestinal health. The higher abundance of GH25 family in Duzang pig’s cecal microbiome of fed *Panax notoginseng* stems and leaves diet. This increase may be the reason for the microbial diversity decrease.

## Introduction

The gastrointestinal tract digests and absorbs nutrients and connects the animal body to its external environment (Awad et al., 2017). The number of microbiomes participating in the host’s nutritional metabolism have colonized it. Enterobacteriaceae, *Clostridium*, *Streptococcus*, and *Enterococcus* predominated in the early establishment of pig intestinal flora (Petri et al., 2010). The abundance of Firmicutes and Spirochetes increased with the age of pigs, but the abundance of Bacteroidetes decreased (Han et al., 2018). During the pigs’growing-finishing stage, Firmicutes, Bacteroidetes, and Proteobacteria are the majority of the gut microbial community (Lamendella et al., 2011). Proteobacteria (70%) and Firmicutes (20%) predominated in the jejunum and ileum of healthy adult pigs, and the cecum and colon were dominated by Firmicutes (35%), Bacteroidetes (21%), Proteobacteria (3%), and Spirochetes (2%; Patil et al., 2020).

Diet composition influenced the colonization and dynamic alterations of intestinal microbiota. “Low-fat, high-fiber” diets stimulate the growth of beneficial flora in pig intestines, with higher abundances of *Lactobacillus*, *Bifidobacteria*, and *Prevotella*, whereas “high-fat, low-fiber” diets may increase Enterobacteriaceae abundance (Heinritz et al., 2016). Dietary fiber helps pigs maintain normal intestinal morphology to balance intestinal flora and enhance intestinal immunity (Jha and Berrocoso, 2015).

The cecum and colon ferment dietary fiber to produce short-chain fatty acids (SCFA), which account for 5% to 24% of the energy required by pig intestinal epithelial cells (Jha et al., 2019). Dietary fiber serves as the fermentation substrate for some intestinal flora. It produces SCFA and other small-molecule metabolites, helping regulate, and maintain intestinal health by preventing the growth of pathogenic bacteria and balancing the flora to enhance intestinal health (Suriano et al., 2022).

The Tibetan pig (Sus scrofa), an ancient breed in China, has been known for its high adaptability to harsh environments (Niu et al., 2022). Tibetan pigs were thought to have good performances of rough feeding tolerance. Duzang pigs are hybrids of Duroc boars and Tibetan sows in Diqing. Before the trial, Duzang pigs were kept grazing, just like Tibetan pigs in Diqing. In recent years, the planting and deep processing of Chinese materia medica have caused a considerable amount of byproducts, which results in resource waste because of a low utilization rate (Duan et al., 2015). *Panax notoginseng* (Burk.) F.H. Chen (Araliaceae) is a well-known, high-value Chinese materia medica with a long history of use, but its stems and leaves are usually discarded (Cicero et al., 2003).

Duzang pigs were used as experimental animals in this study, and they were given feeds containing 0%, 10%, and 20% of *Panax notoginseng* stems and leaves, respectively. Detect the SCFA in the cecal contents. 16S rRNA sequencing was used to analyze the composition, diversity, and relationship of microorganisms in the cecum of Duzang pigs. Metagenomic sequencing for functional annotation of cecal microbes. The study aims to discover the impact of *Panax notoginseng* stems and leaves on the intestinal microbiota of pigs and to provide data support for its application in the diet.

## Materials and Methods

### Animals and Growth Performance Measurement

The Animal Ethics Committee of Yunnan Agricultural University approved all animal experiments. Written informed consent was obtained from the owners for the participation of their animals in this study. Thirty healthy Duzang boars (Duroc pig♂ × Tibetan pigs in Diqing♀) with a body weight of 75.84 ± 7.43 kg were randomly divided into control group (CG), test group I (TGI) and test group II (TGII), each group 10 pigs. The CG group was fed the corn–soybean meal-based diet, and the TGI and TGII groups were fed the diets containing 10% and 20% *Panax notoginseng* stems and leaves, respectively (Supplementary Table S1). The Duzang pigs used in the experiment were bred in Yanshan County, Wenshan Prefecture, Yunnan Province, China. The diet adaptation period was 1 wk, and the test period was 32 d. Each Duzang pig was housed in a separate pen with free access to food and water.

The Duzang pigs in the fasting state were weighed on the first day and day 33, and the average daily gain (ADG) was calculated. ADG = (final weight − initial weight)/32 d. The daily feed weight and remaining feed weight of each pen were recorded, and the average daily feed intake (ADFI) and gain: feed (G: F) were calculated. ADFI = (total amount of feed fed for 32 d − total remaining amount for 32 d)/32 d. G: F = (the total amount of feed consumed in 32 d)/(total weight gain in 32 d).

### Sample Collection and Carcass Trait Measurement

After the feeding experiment, five pigs of each group were fasted for 12 h (with free access to water) and then slaughtered. The cecal contents were collected in 5 mL cryogenic vials and quickly frozen in liquid nitrogen. The samples were stored in a −80 °C refrigerator, and they were used for 16S rRNA sequencing and SCFA detection.

The carcass of Duzang pigs is split along the spine, and the left half is used to measure the carcass traits. The weight of the carcass is obtained after removing the head, tail, hooves, and viscera (retaining the abdominal fat and kidneys). The dressing percentage is calculated by dividing the weight of the carcass by the final body weight. The subcutaneous fat thickness at three points located at the midpoint of the back of the pig at the 6th-7th ribs, the last rib, and the junction of the lumbar vertebrae is measured using a vernier caliper, and the backfat depth is the average of the three measurements.

### Detection of SCFA in Cecal Contents

Vortex a total of 0.1 g of cecal content samples with 1 mL of ultrapure water and centrifuge (12,000 rpm) at 4 °C for 10 min. After centrifugation, take 500 μL supernatant and add 200 μL 25% metaphosphoric acid solution and 300 μL 50% ethanol solution, and centrifuge at 4 °C (12,000 rpm) for 10 min. Then, use a 0.22 μm filter membrane to filter into a sample bottle for testing. Agilent GC6890N (Agilent Technologies, CA, USA) was used to determine the content of acetic acid, propionic acid, and butyric acid in intestinal contents.

Conditions for GC operation: the detector temperature was set to 280 °C, the injection port was set to 250 °C, and the injection volume was 1 μL. Set the column temperature to 70 °C and maintain it for 0.6 min, then raise the temperature to 200 °C and maintain it for 4 min, and finally raise the temperature to 280 °C and maintain it for 5 min. Helium (99.9%) was used as the carrier gas, and the flow rate was 1.0 mL/min. For electron ionization (EI ion source), the ion source temperature was 230 °C; the electron bombardment energy was 70 eV; the quadrupole temperature was set at 150 °C, and the solvent delay was 4 min.

### 16S rRNA Gene Sequencing

The total microbial DNA of the cecal contents was extracted according to the operating instructions of the HiPure Fecal DNA Kit (Magen, China). The total microbial DNA was diluted to 1 μg/μL, and the target sequencing region (V3 + V4) of the 16S rRNA gene was amplified using the diluted DNA as a template. The primer sequences used for amplification were 341F (5ʹ-CCTACGGGNGGCWGCAG-3ʹ) and 806R (5ʹ-GGACTACHVGGGTATCTAAT-3ʹ; Guo et al., 2017). The qualified PCR products after purification and quantitative detection were sequenced, and a gene library was constructed. The high-throughput sequencing platform is Illumina Novaseq 6000 PE250. Use Fastp (version 0.18.0; Chen et al., 2018) software to filter out low-quality reads (including reads with base *N* > 10% and *Q*-value > 20 with base content <50%) and adapters to obtain clean reads. Using Flash (version 1.2.11; Magoč and Salzberg, 2011) software, the paired clean reads were merged and spliced into Tags, the minimum overlap was set to 10 bp, and the mismatch rate was 2%. According to the barcode tag sequence, identify and split the sequencing data of each sample from the tags, and filter the tag to obtain clean tags (Bokulich et al., 2013).

### Microbial Composition and Diversity Analysis

The Uparse algorithm (Edgar, 2013) of Usearch (version 9.2.64) software was used to cluster the clean tags into operational taxonomic units with a default consistency of 97%. The chimeras and Singletons sequences detected during the clustering alignment were removed using the Uchime algorithm (Edgar et al., 2011). According to the principle of the Uparse algorithm, the sequence with the highest abundance in the OTU was selected as the representative sequence of this OTU. Species annotation and classification were performed using the RDP classifier (version 2.2; Wang et al., 2007) based on the SILVA (version 132) database (Pruesse et al., 2007). Indicator species analysis was performed using LDA effect size (LEfSe; version 1.0; Segata et al., 2011). Alpha diversity was analyzed using QIIME (version 1.9.1; Caporaso et al., 2010) and muscle (version 3.8.31) software (Edgar, 2004). Bate diversity was analyzed using FastTree (version 2.1; Price et al., 2010), Vegan package (Oksanen et al., 2012), and GuniFrac package (Lozupone and Knight, 2005).

### Metagenomic Sequencing, Assembly, and Genome Annotation

Using HiPure Fecal DNA Kit (Magen, China) to extract DNA of the three pig’s cecal content samples of each group. After quality inspection, sequencing was on the MGISEQ-2000 platform (BGI, Shenzhen) by combinatorial probe-anchor synthesis technique. Adapters and low-quality reads in raw data were removed using Trimmomatic software (version.0.39; Bolger A et al., 2014). Use Bowtie2 software (version.2.2.23) to align the reads in the valid data with the host genome (Langmead and Salzberg, 2012) and remove the reads on the alignment. Metagenome assembly was performed using the software MEGAHIT (version.1.2.9; Li et al., 2015), and the assembly results were evaluated by QUAST software (Mikheenko et al., 2016).

Contigs below 500 bp were filtered out, and the open reading frames were identified using MetaGeneMark software (version.3.38; Zhu et al., 2010) with default parameters. Redundant genes were removed using CD-HIT software (version.4.8.1; Fu et al., 2012), with a similarity threshold of 95% and a coverage threshold of 90%. DIAMOND software (version.2.0.5) was used to compare non-redundant genes with the Kyoto Encyclopedia of Genes and Genomes (KEGG) and carbohydrate-active enzymes (CAZy) databases (*E* value < 1e^−5^; Ogata et al., 1999; Lombard et al., 2014).

### Basic Statistical Analysis and Graphics Drawing

Data statistical analysis was conducted using SPSS software (version 22.0). The stacked bar plot of the community composition was visualized in the R project ggplot2 package (version 2.2.1). Based on the species abundance table of dominant microbiota in the cecal of Duzang pigs, Spearman rank correlation coefficients were calculated, and correlation network diagrams were drawn using Cytoscape (version 3.8.2). The differences in KEGG and CAZy pathways among groups were computed by the Kruskal–Wallis H test.

## Results

### Growth Performance, Carcass Traits, and Health of Duzang Pigs

The growth performance of Duzang pigs during the 32-d trial is listed in Table 1. The FBW, ADFI, ADG, and G: F had no significant differences in the three groups. The carcass traits between the CG, TGI, and TGII groups with no significant differences (Table 2). All the Duzang pigs in this trial remained healthy.

**Table 1.** Growth performance of Duzang pigs in 32 d

Items	CG	TGI	TGII
FBW, kg	111.28 ± 9.30	112.64 ± 4.73	115.88 ± 10.08
ADFI, kg	2.96 ± 0.40	2.86 ± 0.18	2.97 ± 0.31
ADG, kg	0.60 ± 0.10	0.74 ± 0.06	0.86 ± 0.10
Feed efficiency (G/F)	0.21 ± 0.02	0.26 ± 0.01	0.29 ± 0.01

FBW, final body weight. Data in this table are in the form of mean ± standard error of mean (SEM). Table 2 is the same.

**Table 2.** Carcass traits of Duzang pigs in 32 d

Items	CG	TGI	TGII
Carcass weight, kg	87.16 ± 6.88	88.74 ± 4.33	90.32 ± 9.56
Dressing percentage, %	78.57 ± 1.69	78.69 ± 0.71	77.35 ± 2.06
Carcass straight length, cm	88.00 ± 4.15	88.70 ± 1.54	91.40 ± 2.80
Carcass slanting length, cm	85.40 ± 3.91	87.00 ± 1.30	86.80 ± 3.48
Average backfat depth, cm	4.05 ± 0.16	4.37 ± 0.11	3.94 ± 0.29

### SCFA Concentration in the Cecal Content of Duzang Pigs

**Figure 1.**
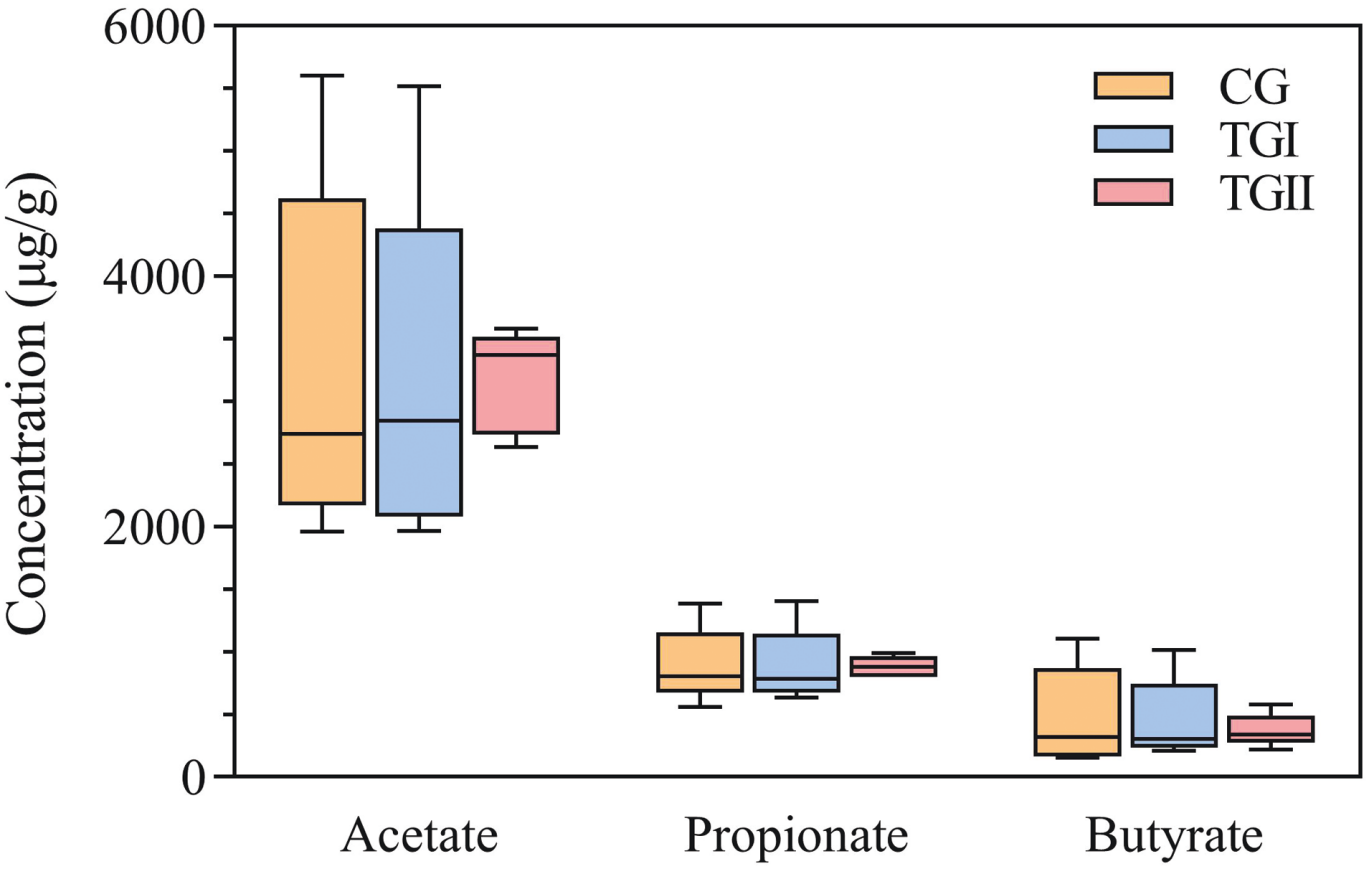
The comparisons of cecal SCFA levels between CG, TGI, and TGII groups of Duzang pigs. The boxplot uses the minimum value to the maximum value and the median as the dividing line.

The concentration of acetate in the cecum of Duzang pigs was higher than that of propionate and butyrate (Figure 1). No significant change was found in the levels of acetate, propionate, and butyrate between the three groups.

### Cecal Microbial Composition and Diversity Analysis by 16S rRNA Sequencing

**Figure 2.**
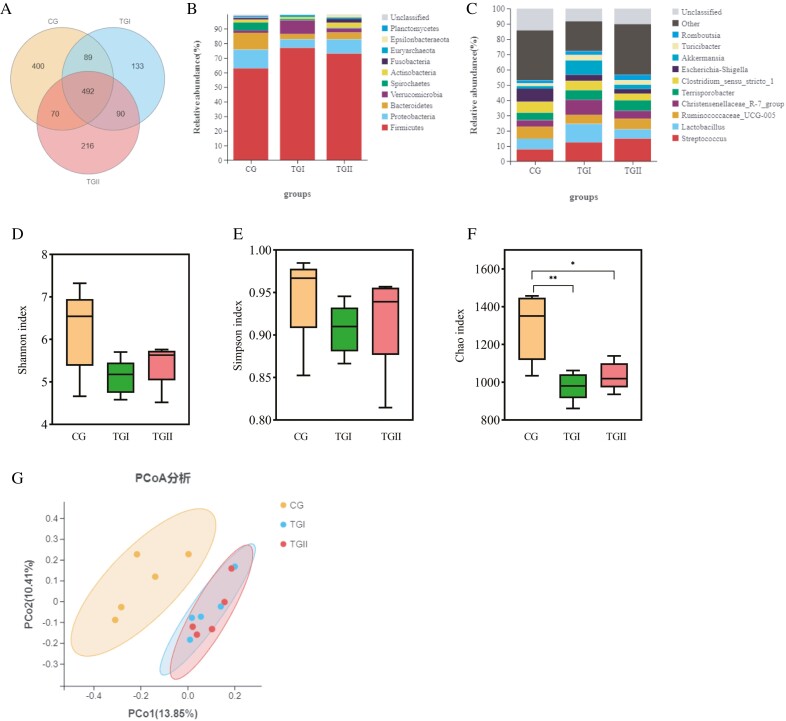
Microbial composition and diversity of the cecal of Duzang pigs with different levels of *Panax notoginseng* stems and leaves (A) OTU analysis, (B) the top 10 bacterial groups with relative abundance at the phylum level, (C) the top 10 flora at the genus-level relative abundance, (D) Shannon index, (E) Simpson index, (F) Chao index, (G) PCoA-graph. *P < 0.05, **P < 0.01.

A total of 1,490 bacterial operational taxonomic units belong to CG, TGI, and TGII groups (Figure 2A). The relative abundance of Firmicutes in the TGI group (76.89%) were higher than that in the TGII group (72.96%) and CG group (62.79%). Bacteroidetes in the TGI group (3.66%) and TGII groups (4.64%) were lower than those in the CG group (11.54%; Figure 2B). The relative abundance of *Streptococcus* in the TGII group (14.88%) was higher than that in the TGI group (12.36%) and CG group (7.83%). The *Lactobacillus*, *Christensenellaceae R 7 group*, and *Akkermansia* in the TGI group (12.14%, 9.87%, and 9.38%) were higher than that in the TGII group (6.03%, 5.12%, and 2.84%) and CG group (6.93%, 4.15%, and 1.70%; Figure 2C).

The alpha diversity of cecal microbiota between the three groups was evaluated. No significant difference in the Shannon index (Figure 2D) and Simpson index (Figure 2E) between the three groups. A significant difference (*P <* 0.05) in the Chao index in the three groups was observed, and the TGI and TGII groups were lower than the CG groups (Figure 2F). The distribution of microbiota between the CG and TGI groups was clustered separately along principal coordinates, and TGI and TGII groups were almost clustered in the same region (Figure 2G).

### Correlation Analysis Between SCFA and Cecal Microbiota

**Figure 3.**
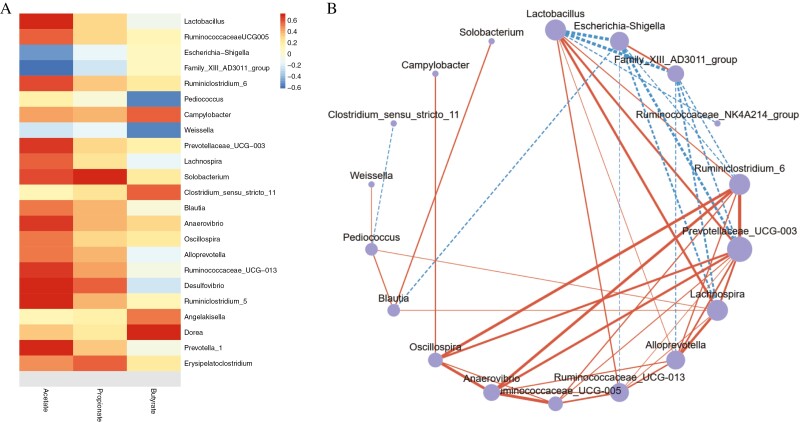
Correlation and abundance analysis of SCFA-related genus. (A) Correlations between SCFA and bacteria at the genus level in the cecal. Spearman’s correlation coefficients are represented by color gradients. Acetate, propionate, and butyrate were related to each microbial species by partial Spearman tests. *P < 0.05, **P < 0.01. (B) Correlation between the bacteria at genus level associated with SCFA. The solid line indicates a positive correlation, while the dotted line indicates a negative correlation. There is a significant difference between those microbiota in Spearman tests (*P <* 0.05).

According to the condition that the abundance of at least one sample is >0.1%, differential genera are screened. A total of 110 screened genera were obtained, Spearman correlation analysis was performed with SCFA, and significantly related items were selected as a heatmap (*P <* 0.05, Figure 3A). We found 14 genera positive associated with acetate, they were *Lactobacillus*, *Ruminococcaceae UCG 005*, *Ruminiclostridium 6*, *Prevotellaceae UCG 003*, *Lachnospira*, *Solobacterium*, *Blautia*, *Anaerovibrio*, *Oscillospira*, *Alloprevotella*, *Ruminococcaceae UCG 013*, *Desulfovibrio*, *Ruminiclostridium 5*, and *Prevotella 1*, *Escherichia Shigella*, and *Family XIII AD3011 group* showed negative correlations. The *Solobacterium*, *Desulfovibrio*, and *Erysipelatoclostridium* were positively associated with propionate. There were positive correlations between butyrate and *Campylobacter*, *Clostridium sensu stricto 11*, *Angelakisella*, and *Dorea*, and negative correlation ships between butyrate and *Pediococcus* and *Weissella*.

Spearman’s correlation analysis was performed on the SCFA-associated genus to reveal entries with significant differences (*P <* 0.05, Figure 3B). The *Escherichia Shigella* and *Family XIII AD3011 group* had positive relationships with each other and showed negative correlations with *Lactobacillus*, *Ruminiclostridium 6*, *Prevotellaceae UCG 003*, and *Lachnospira*. The *Lactobacillus* was positively associated with *Ruminiclostridium 6*, *Prevotellaceae UCG 003*, *Lachnospira*, *Alloprevotella*, and *Ruminococcaceae UCG 013*. The *Ruminiclostridium 6* and *Prevotellaceae UCG 003* were positively associated with *Alloprevotella*, *Ruminococcaceae UCG 005*, *Anaerovibrio*, and *Oscillospira*.

### Selection of Differential Bacterial Communities and Their Correlation With SCFA-Associated Genus

**Figure 4.**
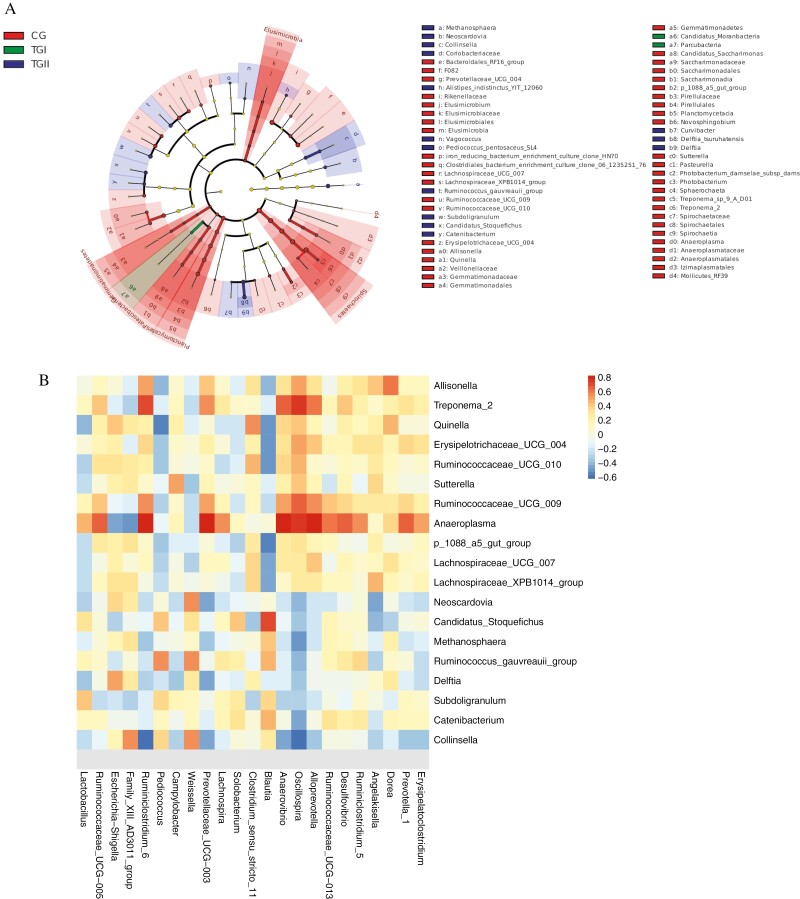
Correlation analysis of gut microbiome related to SCFA concentration in the cecal of Duzang pigs and differential flora. (A) LEfSe analysis explores biomarkers. LDA score (log 10) > 2. (B) Correlations of the SCFA-associated bacteria with the differential flora. *P < 0.05, **P < 0.01.

In the CG group, 47 differential flora were found, which were mainly branches of Bacteroidetes, Spirochaetes, Planctomycetes, Gemmatimonadetes, and Patescibacteria. In the TGI group, two differential flora were found, which were Candidatus Moranbacteria and Parcubacteria. Eleven differential flora were found in the TGII group, which were *Curvibacter*, *Subdoligranulum*, *Methanosphaera*, etc. (Figure 4A).

As exhibited in Figure 4B, we found that two genera had significant positive relationships with the *Ruminiclostridium 6*, *Prevotellaceae UCG 003*, *Anaerovibrio*, *Oscillospira*, and *Alloprevotella*, were *Treponema 2*, *Ruminococcaceae UCG 009* (*P <* 0.05). Moreover, *Weissella* was significantly positively related to *Neoscardovia*, *Ruminococcus gauvreauii group*, and *Collinsella* (*P <* 0.05). The *Anaeroplasma* was significantly positively related to *Prevotella 1* (*P <* 0.05).

### KEGG and CAZy Database Annotation Analysis for Metagenomics

**Figure 5.**
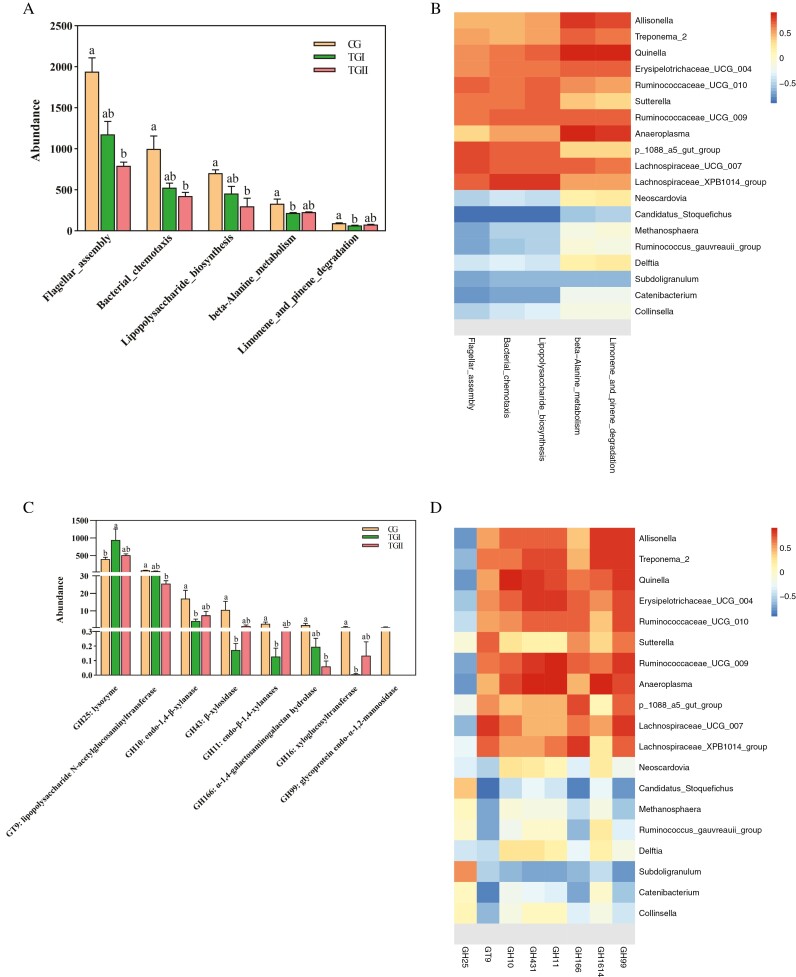
Functional capacities of the cecal microbiome. (A) Function terms of CAZy database. Lowercase shoulders indicate significant differences (*P <* 0.05), the following as the same. (B) Correlations between CAZymes and cecal genus with significant difference. *P < 0.05, **P < 0.01. (C) Function terms of Kyoto Encyclopedia of Genes and Genomes (KEGG) database. (D) Correlations between KEGG pathways and cecal genus with significant difference. *P < 0.05, **P < 0.01.

Using metagenomic sequencing data mapped the microbiota gene catalog onto the KEGG and CAZy modules to identify the function of the cecal microbiome. A total of five KEGG pathways having significantly different abundance were found in three groups (Figure 5A). The flagellar assembly, bacterial chemotaxis, and lipopolysaccharide biosynthesis pathways of the TGII group were significantly lower than the CG group. The beta-alanine metabolism and limonene and pinene degradation pathways of the TGI group were significantly lower than the CG group.

The correlations between KEGG pathway and specific cecal genus were shown (Figure 5B). The *Ruminococcaceae UCG 010*, *p 1088 a5 gut group*, *Lachnospiraceae UCG 007*, and *Lachnospiraceae XPB1014 group* were significantly positive-related to flagellar assembly, bacterial chemotaxis, and lipopolysaccharide biosynthesis pathways, Candidatus Stoquefichus and Catenibacterium were significantly negative (*P <* 0.05). The *Allisonella*, *Treponema 2*, *Quinella*, *Erysipelotrichaceae UCG 004*, *Ruminococcaceae UCG 009*, *Anaeroplasma*, and *Lachnospiraceae UCG 007* were significantly positively related to β-alanine metabolism and limonene and pinene degradation pathways (*P <* 0.05).

Eight CAZymes with significantly different abundances were detected (Figure 5C). The TGI groups had a significantly higher abundance of glycoside hydrolase 25 (GH25) than the CG group (*P <* 0.05). The glycosyl transferase 9 and GH166 in the TGII group were significantly lower than the CG group (*P <* 0.05). The GH10, GH43, GH11, and GH16 in the TGI group were significantly lower than the CG group (*P <* 0.05). The GH99 was not found in the cecal microbiome of TGI and TGII groups.

The *Allisonella*, *Treponema 2*, *Quinella*, *Erysipelotrichaceae UCG 010*, *Ruminococcaceae UCG 009*, *Anaeroplasma*, and *Lachnospiraceae XPB1014 group* were positively associated with six of the GH family, they were GH10, GH143, GH11, GH166, GH16, and GH99, Candidatus stoquefichus and Subdoligranulum were negative (Figure 5D).

## Discussion

SCFA are the end products of the fermentation process by gut microbiota. Previous studies have indicated a close association between Proteobacteria and fecal concentration of SCFA (Primec et al., 2019). *Lactobacillus*, *Bacteroides*, and *Clostridium sensu stricto 1* generate SCFA (Hu et al., 2020). *Catenibacterium* has been shown to increase the production of SCFA by promoting fiber fermentation (He et al., 2018). In a study, a Phytolin + Fiber diet not only affected the microbial composition of pig feces, including an increase in the abundance of *Lactobacillus* and *Catenibacterium*, but also significantly raised the total concentration of SCFA (Loo et al., 2022). The abundance of *Turicibacter*, which synthesizes butyric acid in the gut, is positively correlated with the digestibility of acid-detergent fiber in pigs (Niu et al., 2019). Compared with feeding the basal diet, feeding diets supplemented with 10% or 20% *Panax notoginseng* stems and leaves hardly affected the concentration of SCFA in the cecum. The results showed that the abundance of *Proteobacteria* and *Bacteroides* decreased, but that of *Lactobacillus*, *Christensenellaceae R 7 group*, *Turicibacter*, and *Catenibacterium* increased. This could be due to the microbial community balancing the effects of the diet on the host through changes in relative abundance.

Various bacteria having symbiotic relationships with the host are found in the gastrointestinal tracts of animals (Adak and Khan, 2019). Firmicutes, Bacteroidetes, and Proteobacteria are most prevalent in the adult pig hindgut microbiota (Benson et al., 2010; Perry et al., 2016; Crespo-Piazuelo et al., 2019). In the cecal of Duzang pigs, Firmicutes, Proteobacteria, and Bacteroidetes also predominate. Firmicutes participate in host energy absorption and metabolism. In addition, Bacteroidetes contribute to the host’s carbohydrate, bile acid, and steroid metabolism (Zou et al., 2020). Individuals with a high ratio of Firmicutes/Bacteroidetes have higher energy intake efficiency from food, thereby effectively promoting energy absorption by the host and increasing body weight (Krajmalnik-Brown et al., 2012). In this experiment, feeding diets with 10% and 20% of the stems and leaves of *Panax notoginseng* increased the ratio of Firmicutes to Bacteroidetes in the cecum of Duzang pigs.

*Lactobacillus*, *Christensenellaceae R 7 group*, and *Akkermansia* were detected in the cecal of Duzang pigs. *Christensenellaceae* are thought to be significant potential probiotics for host health as they are found in the intestines of animals and have a considerable inverse correlation with inflammatory diseases (Ganesh et al., 2013). Moreover, it also possesses the ability to degrade plant polysaccharides (Vital et al., 2014). Studies have reported that the relative abundance of *Akkermansia* is positively correlated with mucus thickness and intestinal barrier integrity in animals, and it can produce acetate and propionate, which is beneficial for gut health (Hu et al., 2021, Xia et al., 2021, Wu et al., 2021a). The fecal microbiota transplantation from wild pig to ICR mice and feed low-fiber diet or high-fiber diet changes the relative abundance of *Akkermansia* and *Lactobacillus*, a high-fiber diet with more *Akkermansia* and less *Lactobacillus* (Zhong et al., 2021).

In addition, *Escherichia Shigella* and *Family XIII AD3011 group* had significantly negative correlations with an acetic acid concentration of cecal contents of Duzang pigs, *Pediococcus*, and *Weissella* showed negative correlation ships with butyrate. The *Escherichia Shigella* showed negative correlations with acetic acid, propionic acid, and butyric acid in the feces of pregnant women (Chang et al., 2020). In cecal and colonic contents of rats, negative correlations between the *Family XIII AD3011 group* and acetic acid (Shi et al., 2020). In another study, the *Family XIII AD3011 group* was conducive to improving disease resistance in Tibetan pigs (Shang et al., 2022). *Pediococcus* has been shown to be effective in the production of antimicrobial peptides with applications in the food and health industries (Todorov et al., 2022). *Pediococcus pentosaceus* and *Pediococcus acidilactici* were identified as probiotics (Pupa et al., 2022; Uezen et al., 2023). *Weissella* strains can produce organic acids and have pathogen-inhibitory activities (Teixeira et al., 2021).

The LEfSe analysis revealed a significant increase in the abundance of *Ruminococcus gauvreauii group*, Coriobacteriaceae, *Collinsella*, *Catenibacterium*, and *Neoscardovia* in the cecal of Duzang pigs fed with 20% *Panax notoginseng* stems and leaves diet. Ruminococcaceae is a primary bacterial genus found in the hindgut of animals and is important in the degradation of starch (Wang et al., 2017; Chassard et al., 2021). For instance, *Ruminococcus flavefaciens*, *Ruminococcus albus*, and other related species are widely distributed in degrading plant polysaccharides and producing butyric acid, which provides energy to intestinal epithelial cells and maintains intestinal mucosal integrity (Varel et al., 1984; Biddle et al., 2013). Coriobacteriaceae is associated with glucose metabolism, lipid metabolism, and bile acid metabolism (Karlsson et al., 2013; Clavel et al., 2014). Diet influences the composition of gut microbes. Studies have shown a negative correlation between dietary fiber and the abundance of *Collinsella* (Gomez-Arango et al., 2018). The variations in the intestinal microbiota of pigs from different breeds, characterized by different feed efficiencies, are predominantly associated with *Catenibacterium*, *Clostridium*, and *Bacteroides* (Bergamaschi et al., 2020).

Compositions in metagenomic functional capacities were different in three groups. KEGG comparison analysis demonstrated that the flagellar assembly, bacterial chemotaxis, lipopolysaccharide biosynthesis pathways, beta-alanine metabolism, and limonene and pinene degradation pathways in Duzang pigs cecal microbiome which fed *Panax notoginseng* stems and leaves lower than basal diet. Baicalin-aluminum complexes affected the efficacy of the diarrhea of piglets, KEGG analysis showed the enrichment of flagellar assembly, bacterial chemotaxis, and lipopolysaccharide biosynthesis pathways (Fu et al., 2019). Obese dogs with an increase in Firmicutes and a decrease in Bacteroidetes showed enrichment in flagellar assembly, bacterial chemotaxis, and transport pathways (Thomson et al., 2022). The microbiota of obese resistant rats had enhanced bacterial chemotaxis, phosphotransferase system, and fatty acid biosynthesis compared to obese prone rats whose microbiota had higher glycan degradation and lipopolysaccharide biosynthesis pathways (Obanda et al., 2021). A study suggests that antibiotic-induced changes in gut microbiota might contribute to the inflammation responses through the alternation of metabolic statuses, such as beta-alanine metabolism (Sun et al., 2019). Lipopolysaccharide is associated with gut immunity and microbial dysbiosis (Xiao et al., 2021). The diet containing the *Panax notoginseng* stems and leaves reduces the enrichment of lipopolysaccharide biosynthetic pathway in the cecal microbiome of Duzang pigs, which may have a positive effect on intestinal health.

The CAZyme comparison analysis reflected that GH10 and GH25 were less active in Duzang pigs fed *Panax notoginseng* stems and leaves, and no active GH99 has been found. The family GH10 and GH25, xylan-degrading enzymes, were found in gut Bacteroidetes (Wu et al., 2021b). The GH25 family with peptidoglycan hydrolase activity has the ability to lys other gut microbial cells (Bellinzoni et al., 2014; Moraïs et al., 2016). GH99 family members have been implicated in the ability of Bacteroides spp., present within the gut microbiota, to metabolize fungal cell wall α-mannans, releasing α-1,3-mannobiose by hydrolyzing αMan-1,3-αMan→1,2-αMan-1,2-αMan sequences within branches of the main α-1,6-mannan backbone (Hakki et al., 2015). The decreased relative abundance of Bacteroides in the cecal of Duzang pigs, which fed the diet of *Panax notoginseng* stems and leaves, may be the main reason for the disappearance of bacteria with GH99 activity.

In summary, diets containing *Panax notoginseng* stems and leaves had no effect on the concentration of SCFA in the cecal contents of Duzang pigs but affected the microbial composition and diversity. Firmicutes, Proteobacteria, and Bacteroidetes dominate in the cecal of Duzang pigs. Feeding Duzang pigs with a 10% *Panax notoginseng* stems and leaves diet increases the abundance of *Lactobacillus*, *Christensenellaceae R-7 group*, and *Akkermansia* in the cecal. We also performed functional annotation analysis and found that the *Panax notoginseng* stems and leaves reduce the enrichment of lipopolysaccharide biosynthetic pathway, which may have a positive effect on intestinal health. The higher abundance of GH25 family in Duzang pig’s cecal microbiome of fed *Panax notoginseng* stems and leaves diet. This may be the reason for the decrease in microbial diversity in Duzang pigs fed *Panax notoginseng* stems and leaves.

## Supplementary Material

txad142_suppl_Supplementary_Tables_S1
