# Supplementary material for: Panax notoginseng stems and leaves affect microbial community and function in cecum of duzang pigs
Source: Transl Anim Sci. 2024 Jan 22;8:txad142. doi: 10.1093/tas/txad142 (PMC10904106; doi:10.1093/tas/txad142)
Supplement: txad142_suppl_Supplementary_Tables_S1 [file txad142_suppl_supplementary_tables_s1.docx]

Table S1 Composition of basal diet and experimental diet

| Ingredients (%) | CG | TGI | TGII |
| --- | --- | --- | --- |
| Corn | 72.10 | 62.50 | 46.20 |
| Soybean meal | 17.40 | 18.40 | 18.80 |
| Wheat bran | 6.00 | 0.00 | 0.00 |
| Soybean oil | 0.00 | 4.60 | 10.50 |
| *Panax notoginseng* stems and leaves | 0.00 | 10.00 | 20.00 |
| Salt | 0.50 | 0.50 | 0.50 |
| Premix^#^ | 4.00 | 4.00 | 4.00 |
| Nutritional level | | | |
| Digestible energy (MJ/kg) | 13.35 | 13.49 | 13.62 |
| Crude protein | 14.86 | 14.85 | 14.86 |
| Lysine | 0.67 | 0.64 | 0.62 |
| Calcium | 0.58 | 0.59 | 0.59 |
| Total Phosphorus | 0.50 | 0.52 | 0.51 |

^#^: Provide for pigs: VA 2200 IU, VD 700 IU, VE 14 IU, VB6 1.50 mg, VB12 12 µg, VK3 0.55 mg, folic acid 0.45 mg, VB1 11 mg, biotin 0.05 mg, VB2 2.50 mg, pantothenic acid 10.00 mg, niacin 10 mg, choline chloride 350 mg, selenium 0.25 mg, copper 8 mg, iron 70 mg, zinc 50 mg, manganese 10 mg, iodine 0.35 mg.
